# The Dissociation of Latrophilin Fragments by Perfluorooctanoic Acid (PFOA) Inhibits LTX^N4C^-Induced Neurotransmitter Release

**DOI:** 10.3390/toxins17070359

**Published:** 2025-07-20

**Authors:** Evelina Petitto, Jennifer K. Blackburn, M. Atiqur Rahman, Yuri A. Ushkaryov

**Affiliations:** 1 Medway School of Pharmacy, University of Kent, Chatham ME4 4TB, UK; evelina.petitto@ashfiedmedcomms.com (E.P.); jennifer.blackburn@yale.edu (J.K.B.); 2 Department of Life Sciences, Imperial College London, London SW7 2AZ, UK

**Keywords:** α-Latrotoxin, LTX^N4C^, exocytosis, neurotransmitter release, neuromuscular junction, ADGRL1, latrophilin-1, perfluorooctanoic acid (PFOA), inhibition

## Abstract

α-Latrotoxin stimulates neurotransmitter release by binding to a presynaptic receptor and then forming ion-permeable membrane pores and/or stimulating the receptor, latrophilin-1, or Adhesion G-protein-coupled receptor type L1 (ADGRL1). To avoid pore formation, we use the mutant α-latrotoxin (LTX^N4C^), which does not form pores and only acts through ADGRL1. ADGRL1 is cleaved into an N-terminal fragment (NTF) and a C-terminal fragment (CTF), which behave as independent cell-surface proteins, reassociating upon binding LTX^N4C^. We investigated the role of the NTF-CTF association in LTX^N4C^ action, using perfluorooctanoic acid (PFOA). We demonstrate that at low concentrations (≤100 μM) PFOA does not adversely affect ADGRL1-expressing neuroblastoma cells or inhibit LTX^N4C^ binding. However, it causes the dissociation of the NTF-CTF complexes, independent redistribution of the fragments on the cell surface, and their separate internalization. PFOA also promotes the dissociation of NTF-CTF complexes induced by LTX^N4C^ binding. When applied to mouse neuromuscular junctions, PFOA inhibits LTX^N4C^-induced neurotransmitter release in a concentration-dependent manner. Our results indicate that ADGRL1 can mediate LTX^N4C^ signaling only while its fragments remain associated. These findings explain some aspects of receptor-dependent toxin action and contribute to a mechanistic understanding of ADGRL1 functions in neurons.

## 1. Introduction

α-Latrotoxin (αLTX) from black widow spider venom is a powerful presynaptic neurotoxin that causes exhaustive release of neurotransmitters from various mammalian neurons and neurosecretory cells [1,2,3]. Consequently, the toxin has been widely utilized to study the mechanisms of neurotransmitter release [4,5,6]. However, many details of its mechanism of action remain enigmatic and require additional research [7].

For its action, the toxin requires a receptor located in the presynaptic membrane [8,9]. Upon binding to the receptor, the toxin exerts its effects through two distinct mechanisms: the formation of Ca^2+^-permeable pores in the presynaptic membrane and the activation of the receptor [9,10]. The pore’s permeability to Ca^2+^ has made it challenging to fully understand the receptor’s role in αLTX action. However, an αLTX mutant (LTX^N4C^), developed and named by Thomas Südhof [11], lacks the ability to form pores while retaining the ability to activate the receptor and induce neurotransmitter release [12,13,14,15]. This invaluable tool has facilitated the study of the role of receptors in regulating exocytosis [16].

Several neuronal proteins have been shown to bind αLTX: neurexin Iα (NRX) [17,18], latrophilin 1 [19,20,21,22], and receptor-like protein tyrosine phosphatase σ 1 (RPTPσ) [23]. Latrophilin 1 is a member of the family of adhesion G-protein-coupled receptors (AGPCRs). According to the IUPHAR nomenclature, it is also known as AGPCR type L1 (ADGRL1). Of the three αLTX-binding proteins, only ADGRL1 meets all the criteria of a functional LTX^N4C^ receptor [9]: it binds the toxin in the absence of Ca^2+^ [12], localizes in the presynaptic membrane [24], mediates toxin-induced intracellular Ca^2+^ signaling when introduced into non-neuronal cells [25], and is strictly necessary for LTX^N4C^-induced synapse activation [26].

**Figure 1:**
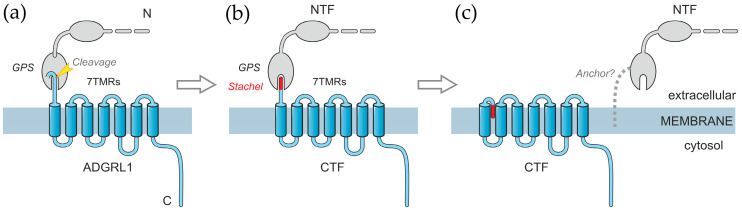
A scheme of ADGRL1 structure and fragmentation. (**a**) The receptor is synthesized as a long protein with a large glycosylated extracellular N-terminal domain (shown partially) and a 7TMR C-terminal domain. During intracellular maturation, ADGRL1 cleaves itself within the GPS domain. (**b**) The cleavage creates two fragments, the NTF and CTF, which remain non-covalently associated, with the N-terminal peptide of the CTF (*stachel*) being lodged in a pocket inside the GPS domain. (**c**) As a result of a poorly understood process, the NTF dissociates from the CTF on the cell surface, while still remaining anchored in the membrane by an unknown mechanism. When it is released from the GPS domain, the hydrophobic *stachel* peptide of the CTF buries itself in a conserved cavity within the 7TMRs, where it also interacts with the extracellular loop 2 [30,31]. This activates the CTF, leading to its interaction with a G-protein and subsequent intracellular signaling [30,31], described in Discussion.

ADGRL1 is a typical AGPCR with a long adhesion-like extracellular N-terminal domain, seven transmembrane regions (7TMRs), and a long intracellular C-terminal tail (Figure 1a) [21,22]. During its intracellular processing, ADGRL1 undergoes autocleavage at the G-protein-coupled receptor proteolysis site (GPS, also known as the GAIN domain [27]). This produces two distinct fragments [28]: the extracellular N-terminal fragment (NTF) and the intramembrane C-terminal fragment (CTF) (Figure 1a,b). The NTF, which lacks transmembrane domains, was thought to be retained on the cell surface due to its non-covalent interaction with the CTF [28], which involves the N-terminal peptide of the CTF (*stachel*, spike in German) and the GPS domain (Figure 1b). The two fragments of AGPCRs have distinct functions: the NTF interacts with soluble ligands, the extracellular matrix, and/or surface proteins of other cells [29], whereas the CTF binds G-proteins and mediates ligand-induced signals that trigger exocytosis [29,30].

Surprisingly, several studies suggest that the NTF can separate from the CTF while remaining attached to the cell surface by an unknown anchor [16,25,32] (Figure 1c). Furthermore, the fragments can be separated within the membrane by antibody-induced patching of the NTF, and the two fragments undergo separate endocytosis [16]. This suggests that the NTF and CTF can behave as independent cell-surface proteins with different functions. Notably, the binding of a ligand, like LTX^N4C^ or LASSO/teneurin-2, to the NTF facilitates the reassembly of these two fragments [7,25], thereby triggering signaling [16,25,26]. Replacing the CTF in recombinant ADGRL1 with a non-signaling moiety inhibits intracellular Ca^2+^ signals [16,25], suggesting that NTF-CTF reassembly is essential for intracellular signaling. However, it remains unclear whether, in a native synapse, a ligand can induce signaling through the NTF alone, without its reassociation with the CTF.

To develop a biochemical tool for separating the native NTF and CTF, we considered using perfluorooctanoic acid (PFOA). PFOA is a mild, non-lipophilic surfactant capable of solubilizing multi-subunit proteins without causing their dissociation into individual subunits [33,34]. Also, prolonged exposure to PFOA concentrations below 200 μM does not negatively impact cultured cells [35,36], indicating that this detergent does not damage cell membranes. PFOA specifically binds human serum albumin at a high-affinity site without disrupting its secondary structure [37]. On the other hand, PFOA was demonstrated to differentially extract the NTF from ADGRL1-expressing cells [16], suggesting that this detergent can disrupt NTF-CTF complexes by interacting specifically with one or both fragments and thus can be utilized to study the interactions between the ADGRL1 fragments in live cells.

In this paper, we examined the impact of low concentrations of PFOA on ADGRL1 fragments and the biological action of LTX^N4C^. Our findings indicate that 100 μM PFOA induces the dissociation of ADGRL1 fragments on the cell surface and blocks the burst-like transmitter exocytosis induced by LTX^N4C^ in motor neurons. This supports the hypothesis that LTX^N4C^ activity requires the reassociation of ADGRL1 fragments.

## 2. Results

### 2.1. PFOA Disrupts the NTF-CTF Complexes

PFOA was previously shown to solubilize the NTF of a recombinant ADGRL1 construct expressed in neuroblastoma cells, while leaving the CTF in the membrane [16]. To determine whether a similar effect can also be observed in native synapses, we treated rat brain synaptosomal plasma membranes with varying concentrations of PFOA at different temperatures. In parallel, we employed varying concentrations of Thesit, a traditional non-ionic detergent typically utilized to solubilize ADGRL1 while maintaining its ligand-binding ability. After detergent treatment, the membranes were centrifuged and analyzed by Western blotting to determine the NTF and CTF distribution between the supernatant and pellet (unsolubilized membranes). Concurrently, the fractions were tested for two other membrane proteins: the potassium channel (Kv1.2), which contains four TMRs, and NRX, which possesses a single TMR.

**Figure 2:**
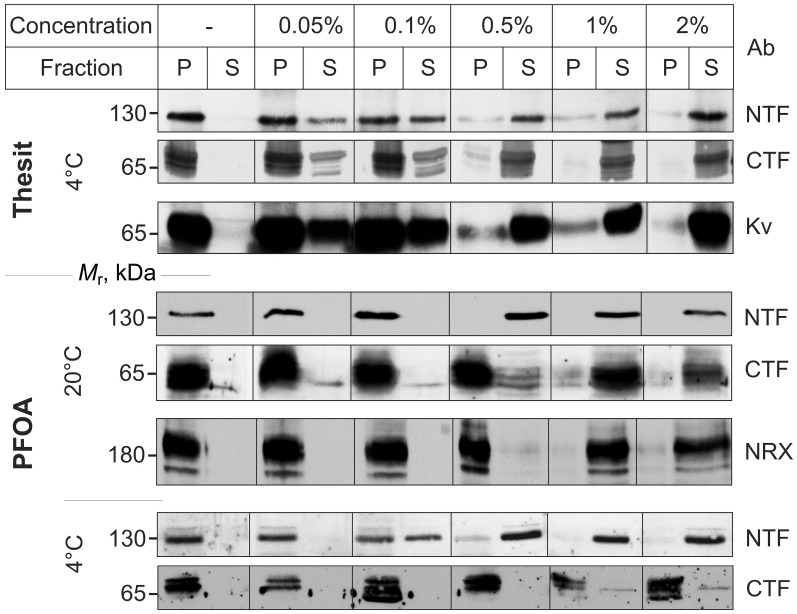
Differential solubilization of the neuronal NTF and CTF by increasing concentrations of Thesit or PFOA at different temperatures. Equal samples of rat brain synaptosomal plasma membranes were treated with different concentrations of detergents (as indicated at the top) at 4 °C or 20 °C (as shown on the left). After centrifugation, aliquots of the supernatants (S) and pellets (P) were analyzed by Western blotting using antibodies against the NTF, CTF, potassium channel (Kv), or NRX. The blots are representative of *n* = 3 experiments, which produced similar results. Note that Thesit progressively solubilizes equal proportions of the NTF, CTF, and Kv1.2, whereas PFOA differentially extracts the NTF, leaving 95% of the CTF in the membrane at all concentrations at 4 °C.

Figure 2 shows that Thesit began extracting both ADGRL1 fragments in equal proportions at a concentration of 0.05% and fully solubilized them at 1%. Thesit also solubilized Kv1.2 in a similar manner. In comparison, PFOA solubilized the NTF and CTF differentially. At 20 °C, 0.5% PFOA completely extracted the NTF but only a small fraction of the CTF and NRX. The latter required at least 1% PFOA for successful solubilization by PFOA. The differential solubilization was even more prominent at 4 °C: PFOA partially extracted the NTF from the membrane at 0.1% concentration and fully at 1%, while the CTF remained within the membrane at all PFOA concentrations (with the exception that a small amount of one phosphorylated form of the CTF [38] was also extracted by 1–2% PFOA).

Another reported finding related to the effects of PFOA was that αLTX, a functional ADGRL1 ligand, can counteract the disruptive effect of PFOA and enable the co-purification of the CTF with the NTF during αLTX-affinity chromatography [16]. To avoid the impact of a functional ligand, we examined the NTF-CTF interaction by affinity chromatography on wheat germ agglutinin (WGA). WGA binds to the carbohydrate moiety of the NTF but does not stimulate exocytosis [19,39] and is, therefore, not expected to promote NTF-CTF complex formation. It should also be noted that WGA does not bind the CTF, which is not glycosylated [38].

**Figure 3:**
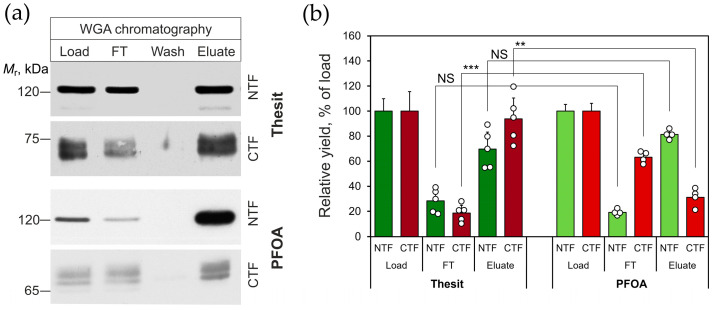
Low concentrations of PFOA inhibit the NTF-CTF reassociation. (**a**) Affinity chromatography of native ADGRL1 using WGA as an affinity ligand. Rat brain synaptosomes were solubilized in 2% Thesit or PFOA, diluted to 0.04% (100 μM) detergent, passed through a WGA column, and eluted by heating with 1% SDS to ensure complete elution of all bound proteins. Each fraction was then analyzed by Western blotting using anti-NTF and anti-CTF antibodies, as indicated. The blots shown are representative of *n* = 4 experiments; FT, Flow Through. (**b**) Quantification of the affinity chromatography results by computer-aided densitometry of Western blots as in (**a**). The bars are the means ± SE; open circles are individual data points; symbols denote statistical significance of the difference between indicated values: **, *p* < 0.01; ***, *p* < 0.001; *NS*, non-significant; *n*, *N* = 4.

Figure 3a demonstrates that both the NTF and the CTF of ADGRL1 were purified on a WGA-affinity column in both Thesit and PFOA. The column bound the NTF similarly in Thesit (70 ± 13% of load) and PFOA (81 ± 1% of load). However, the proportion of the CTF co-purifying with the NTF differed significantly between the Thesit and PFOA chromatographies. Nearly all (94 ± 10%) of the CTF loaded in Thesit was retained by the WGA-bound NTF (Figure 3b). In contrast, of all the CTF loaded in PFOA, only 31 ± 8% remained bound to the NTF on the column (Figure 3b).

Collectively, these data suggest that 0.1–0.5% PFOA is capable of fully separating the NTF from CTF by selectively solubilizing the NTF. Moreover, even at a lower concentration (0.04%) that itself does not cause NTF solubilization (Figure 2), PFOA can disrupt at least 70% of the NTF-CTF complexes (Figure 3). Considering that WGA could have some positive impact on the NTF-CTF reassociation during the chromatography, it is possible that 0.04% PFOA is more capable of disrupting complexes than follows from Figure 3. Consequently, these experiments demonstrate that PFOA can effectively dissociate the NTF-CTF complexes without solubilizing the membrane and thus can be used to dissect the role of the NTF-CTF interaction in exocytosis induced by LTX^N4C^.

### 2.2. PFOA Induces NTF and CTF Redistribution in Cell Membrane

Prior to testing the effect of PFOA on the LTX^N4C^ mechanism of action, we investigated how PFOA might affect the cell membrane, toxin binding to the receptor, and/or the interaction between the ADGRL1 fragments on the cell surface.

**Figure 4:**
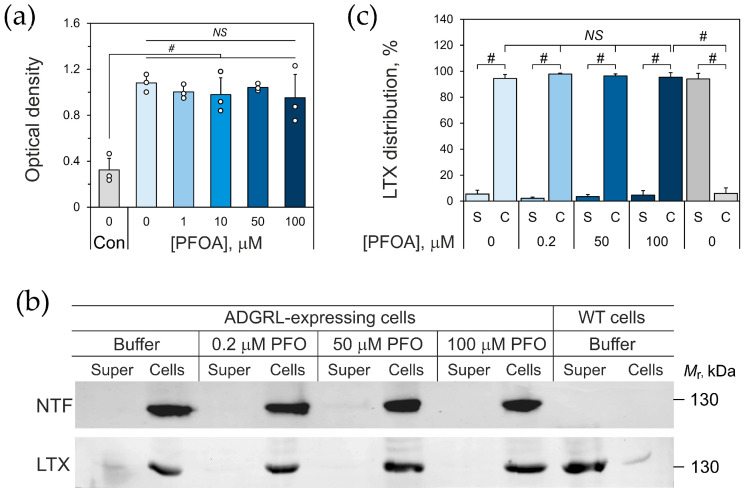
Low concentrations of PFOA do not damage the cell membrane and do not inhibit αLTX binding. (**a**) MTT-based cell viability test. The wells contained ~10,000 cells; no cells were added to control wells. The number of independent experiments *n* = 3, with 4 replicates in each (*N* = 12). Note that 4 h incubation with up to 100 μM PFOA does not significantly affect the viability of cells. (**b**) An immunoblot of ADGRL1-expressing NB2a cells, which were incubated with 0–100 μM PFOA and/or 5 nM αLTX and centrifuged; the resulting supernatants (Super) and cells (Cells) were analyzed by immunoblotting with antibodies against the NTF and αLTX, as indicated. Wild-type (untransfected) NB2a cells were processed in parallel as a control of LTX binding specificity and gel loading. Note that αLTX binds specifically to ADGRL1, and PFOA does not inhibit this binding. (**c**) Quantification of αLTX binding to the ADGRL1-expressing cells using immunoblots as in (**a**). S, supernatants; C, cells. The blot is representative of four independent experiments (*n* = 4), all of which gave similar results. The graph bars are the means ± SE; open circles (where shown) are the means from 3 independent experiments; the symbols denote the statistical significance of the difference between indicated values: #, *p* < 0.0001; *NS*, non-significant.

Previous publications demonstrated that, at concentrations below 300 μM, PFOA is not toxic to cultured cells [36], and an MTT test of cell viability after 4–24 h of treatment with 1–100 μM PFOA fully confirmed these observations (Figure 4a and Figure S1a). As an additional approach to assessing cell viability, we used the propidium iodide (PI) assay [40,41]. As demonstrated in Figure S1b, cell death induced by a 24 h incubation with PFOA was only detectable at concentrations above 2 mM, which is 20 times higher than the highest PFOA concentration used in our experiments. Finally, we evaluated cells’ morphology and ability to proliferate after treatment with 100 μM PFOA and found no difference from control cell cultures (Figure S1c).

To exclude any potential interference of PFOA with LTX^N4C^-ADGRL1 interaction in subsequent physiological experiments, we tested toxin binding to receptor-expressing NB2a cells in the presence of 0.2–100 μM PFOA. As shown in Figure 4b,c, αLTX specifically bound to the receptor-expressing cells only, and this binding was not affected by any of the PFOA concentrations used in these experiments.

**Figure 5:**
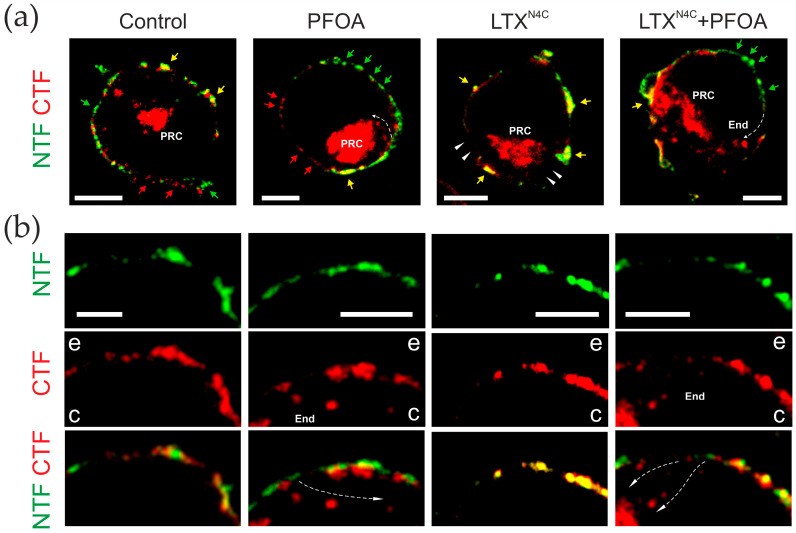
PFOA causes the dissociation of NTF–CTF complexes and the redistribution of individual fragments within the cell. ADGRL1-expressing NB2a cells were incubated for 20 min with 100 μM (0.04%) PFOA and/or 5 nM LTX^N4C^, fixed, permeabilized, and immunostained using fluorescent secondary antibodies for the NTF (green) and CTF (red) (see Section 4.1 and Section 4.3 for details). The cells were then imaged under a confocal microscope. (**a**) Representative 1 μm thick equatorial optical sections of the whole cells treated and stained as indicated. End, endosomes; PRC, perinuclear endocytic recycling compartment; colored arrows point to membrane areas containing the NTF only (green), the CTF only (red), or both fragments colocalized (yellow); white arrowheads show the areas devoid of both ADGRL1 fragments after LTX^N4C^ treatment; dotted arrows indicate CTF endocytosis; scale bar, 5 μm. (**b**) Representative magnified confocal images of small segments of the plasma membrane of cells stained and treated as in (**a**), showing the typical changes in fragment distribution and internalization. Scale bars, 3 μm; CTF endocytosis pathways are indicated by dotted lines with arrows; c, the cytosol; e, extracellular space. All images are representative of *n* = 3 independent experiments, with 6–17 cells imaged for each condition (*N* = 18–51).

**Figure 6:**
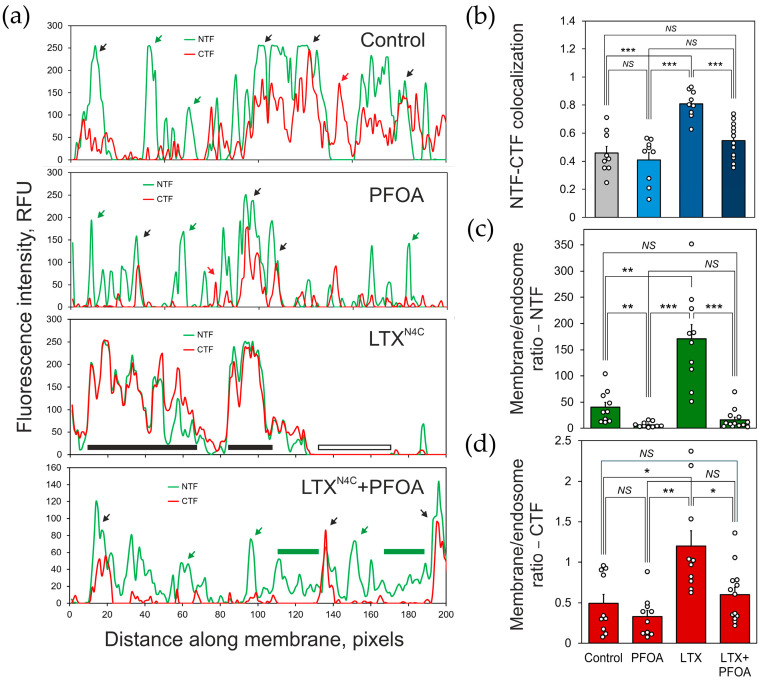
Analysis of the redistribution of NTF-CTF complexes and internalization of the CTF in cells treated with PFOA with or without LTX^N4C^. (**a**) Representative profiles of the NTF and CTF fluorescence intensity distribution along the plasma membrane under indicated conditions, obtained by confocal microscopy as in Figure 5 (see Section 4.5 for details). *RFU*, relative fluorescence units; green line, NTF fluorescence intensity; red line, CTF fluorescence intensity; black arrows, membrane sites where the fragments colocalize; green arrows, the NTF is present only; red arrow, the CTF is present only; black bars, membrane areas with high NTF-CTF colocalization; empty bar, a membrane area devoid of both fragments; green bars, membrane areas occupied by the NTF only. (**b**) The colocalization (Pearson’s correlation coefficient *r*) of the NTF and CTF in the cell membrane under respective conditions, quantified using the fluorescence distribution profiles as in (**a**). (**c**,**d**) Changes in the distribution of the NTF (**c**) and CTF (**d**) between the cell membrane and the endosomal compartments caused by respective treatments. The graph bars are the means ± SE, the open circles are individual data points; the symbols denote the statistical significance of the difference between connected values: *, *p* < 0.05; **, *p* < 0.01; ***, *p* < 0.001; *NS*, non-significant; *n* = 3, with 6–17 images quantified for each condition (*N* = 18–51). Note that LTX^N4C^ substantially increases the colocalization of the NTF and CTF, while PFOA treatment lowers their colocalization by causing the CTF dissociation and internalization.

These observations ensured that it was possible to examine the impact of a non-toxic PFOA concentration (100 μM) on the distribution of the ADGRL1 fragments on the surface of receptor-expressing cells. NB2a clonal cells stably transfected with ADGRL1 (see Section 4.3 for details) were incubated with 100 μM PFOA and/or 5 nM LTX^N4C^ and then immunostained for the NTF and CTF, as described in the methods and in the legend to Figure 5. The confocal fluorescence images of the treated cells are shown in Figure 5, while Figure 6 presents the analysis of any changes in fragment colocalization and distribution.

Consistent with previous publications [16,25], the distribution of the NTF and CTF in the plasma membrane of control (untreated cells) was uneven (Figure 5) and demonstrated a relatively low level of colocalization (Pearson’s correlation coefficient ~0.45) (Figure 6a,b and Figure S2), with small patches containing either the NTF or the CTF only. Interestingly, most of the NTF (~97.6%) was found on the plasma membrane, with only a small proportion (~2.4%) being dispersed around the cytosol, most likely in the endoplasmic reticulum. By contrast, about 66.7% of the CTF was localized inside the cell, in distinct vesicular organelles morphologically identified as endosomes at various maturation stages (End) and perinuclear endocytic recycling compartments (PRCs) near the microtubule organizing center. These vesicles did not contain any NTF, and, in general, no colocalization of the intracellular NTF and CTF was observed. The proportion of the CTF on the cell surface, on average, constituted about 33.3% of its total amount in the cell, suggesting the propensity of the CTF to undergo internalization and recycling, characteristic of many G-protein-coupled receptors after they undergo activation. This observation further confirmed the ability of the NTF, which possesses no transmembrane domains, to anchor in the membrane independently of the CTF.

Treatment with 100 μM PFOA did not significantly affect the low degree of NTF-CTF colocalization on the cell surface (correlation coefficient ~0.41) but increased the length of plasma membrane areas occupied by the NTF alone (Figure 5b, Figure 6b, and Figure S2a,b). PFOA also induced the internalization of some NTF but mostly CTF (Figure 5, Figure 6c,d and Figure S2b,c), which relocated from the NTF-rich areas into End and PRCs along pathways that were traceable as rows of endocytosed vesicles (indicated by dotted arrows in Figure 5), leaving only ~24% of the surface-exposed CTF.

In the absence of PFOA, 5 nM LTX^N4C^ caused an extensive aggregation of the NTF and CTF into large patches on the cell surface (Figure 5), strongly increasing the colocalization of these fragments (correlation coefficient ~0.81) (Figure 5, Figure 6b, and Figure S2a,b). The toxin also triggered the recycling of both the NTF and CTF from intracellular compartments to the plasma membrane (Figure 5, Figure 6c,d, and Figure S2c). As a result, ~99% of all the NTF and ~55% of the CTF appeared on the surface of the cell.

When LTX^N4C^ was applied after treating cells with PFOA, it slightly increased the size of NTF-CTF patches on the plasma membrane (Figure 5b) without significantly increasing overall colocalization of the fragments (correlation coefficient ~0.54) (Figure 6b and Figure S2). It also failed to significantly promote the recycling of the intracellular CTF to the cell surface (37% of all the CTF in the plasma membrane) (Figure 6c,d and Figure S2c).

The Costes statistical significance test [42], which yielded a value of 1 for all colocalization measurements conducted in this study, indicated that the observed values of the colocalization coefficients—namely Pearson’s correlation *r* (Figure 6b,d and Figure S2b) and Manders’ split coefficients (Figure S2c)—were significantly higher than those expected under conditions of random overlap between the two fragments.

Based on these data we proposed that PFOA causes the dissociation of the NTF-CTF complexes, which is anticipated to disrupt (or hinder the formation of) functional receptors and inhibit the generation of an intracellular signal that typically contributes to LTX^N4C^-induced transmitter exocytosis.

### 2.3. PFOA Inhibits LTX^N4C^-Induced Neurotransmitter Release

To test this hypothesis, we investigated how low concentrations of PFOA influence acetylcholine release at the neuromuscular junctions (NMJs), a well-characterized target synapse of LTX^N4C^ [2,12]. Recordings of spontaneous miniature endplate potentials (MEPPs) were conducted on mouse neuromuscular preparations, which were incubated with 0–100 μM PFOA and then stimulated with 0.25 nM LTX^N4C^.

**Figure 7:**
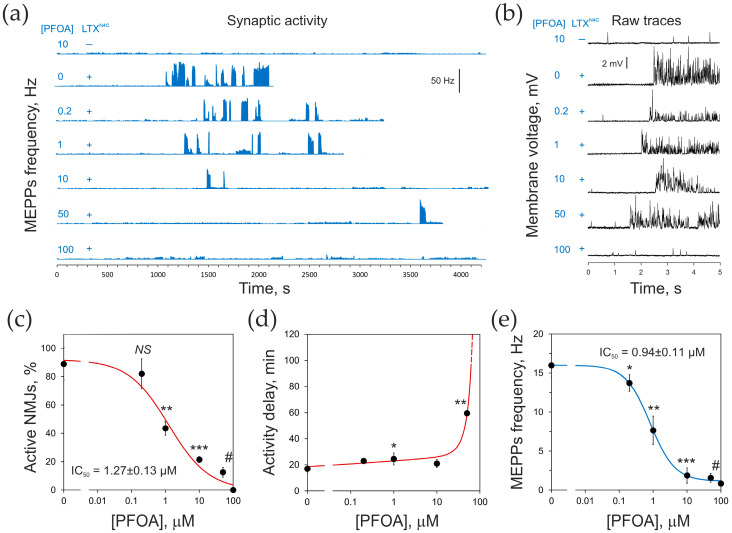
Pretreatment with PFOA inhibits LTX^N4C^-induced synaptic activity. Mouse neuromuscular preparations were incubated for 20 min in increasing concentrations of PFOA and stimulated by 0.25 nM LTX^N4C^ at time 0, as shown on the left for (**a**) and (**b**). NMJ activity was recorded postsynaptically as the frequency of MEPPs. (**a**) MEPP frequency under respective conditions, binned into 1 s intervals. Sharp increases in MEPP frequency form clearly identifiable bursts. Note the delay in the onset of synaptic activity, which is characteristic of LTX^N4C^ action, and the decrease in the number of bursts caused by the increasing [PFOA]. (**b**) Sample raw recordings of NMJ activity demonstrating LTX^N4C^-induced bursts of MEPPs observed at all PFOA concentrations, except 100 μM. (**c**–**e**) Parameters of LTX^N4C^-induced presynaptic activity as a function of the PFOA concentration: the percent of NMJs tested that showed any LTX^N4C^-induced bursts of MEPPs (**c**); the lag-time in the onset of MEPP bursts (**d**), and the overall frequency of MEPPs (**e**). The data points in (**c**–**e**) are the means ± SE; the asterisks denote the statistical significance of respective data points compared to 0 μM PFOA: *, *p* < 0.05; **, *p* < 0.01; ***, *p* < 0.001; #, 0.0001; *NS*, non-significant; *n* = 3–6, *N* = 16–32 individual synapses recorded. IC_50_ values calculated from the dose–response curves are shown in panels (**c**) and (**e**).

The average MEPP frequency in control (untreated) NMJs was 0.56 ± 0.14 Hz and the average MEPP amplitude was 0.82 ± 0.11 mV (Figure S3a). PFOA at any concentration did not significantly change the rate of spontaneous exocytosis or the average MEPP amplitude, which in 10 μM PFOA were 0.69 ± 0.38 Hz and 0.98 ± 0.05 mV, respectively (Figure 7a, trace 1; Figure S3a).

As was previously demonstrated [2], in untreated NMJs, the mutant toxin induced a very characteristic burst-like increase in the frequency of MEPPs (Figure 7a, trace 2). The bursts of exocytotic activity (Figure 7a,b) continued for more than 1 h and were interspersed with short periods of low-frequency MEPPs. The length of an average high-frequency burst was 25–30 s. The frequency of MEPPs in a burst varied and on average was 42.5 ± 12.4 Hz (Figure S3b), which is ~62 times higher than in the control. Despite its strong effect on the rate of spontaneous exocytosis, the toxin did not change the average MEPP amplitude (Figure S3b), which indicates that its action was purely presynaptic, affecting the frequency of vesicle fusion events rather than any postsynaptic processes.

Another notable characteristic of the toxin-induced neurotransmitter release, observed with both the wild-type αLTX and LTX^N4C^, is a significant delay before the onset of their activity. On average, 0.25 nM LTX^N4C^ stimulated control NMJs after a lag-time of ~17 ± 2.5 min.

Pretreatment of neuromuscular preparations with PFOA affected the LTX^N4C^-induced rate of exocytosis in several ways and in a concentration-dependent manner. First, the proportion of NMJs responding to stimulation with LTX^N4C^ by showing high-frequency bursts significantly decreased with increasing concentrations of PFOA (IC_50_ = 1.27 ± 0.13 μM) (Figure 7a,c). NMJs treated with 100 μM PFOA did not demonstrate any bursts of MEPPs (Figure 7a,c, bottom traces). Second, the lag-time before the onset of LTX^N4C^ activity greatly increased with increasing PFOA concentration (Figure 7a,d) and was never achieved within the experiment (3 h) at 100 μM PFOA (Figure 7a,d). As a result of these PFOA-induced changes in LTX^N4C^-triggered synaptic activity, the overall frequency of acetylcholine release decreased exponentially with increasing PFOA concentration, reaching the value of unstimulated control at 100 μM PFOA (IC_50_ = 0.94 ± 0.11 μM) (Figure 7e).

Notably, none of the PFOA concentrations affected the amplitude of LTX^N4C^-induced high-frequency MEPPs (Figure S3a). Furthermore, under all conditions where the LTX^N4C^-induced bursting activity was observed, the average frequency of exocytosis within bursts (Figure 7a,b) did not differ significantly from the control condition (in the absence of PFOA) (Figure S3b). This indicates that the inhibitory effect of PFOA did not modify the mechanism of LTX^N4C^ action but simply decreased its efficiency.

This hypothesis suggested that if PFOA is added after the LTX^N4C^ activity develops, it should be able to dissociate the functional receptors that are being activated by the toxin and thus inhibit or block its characteristic signaling. In such a reverse experiment, 100 μM PFOA was added after LTX^N4C^ had triggered high-frequency bursts, and this stopped the toxin-specific effect almost entirely within 2 min (Figure S4), demonstrating a fast mechanism of action of PFOA.

## 3. Discussion

The goal of this study was to demonstrate that the αLTX receptor ADGRL1 can only mediate the toxin’s effect on neurotransmitter exocytosis if its fragments associate and form a complete, functional GPCR. This appears to be a self-evident conclusion because many researchers believe that the NTFs of AGPCRs are anchored on the cell surface through their strong non-covalent interaction with respective CTFs; the two fragments represent subunits of a complete receptor and, therefore, do not need to undergo any additional reassociation.

However, multiple publications have challenged this view by demonstrating that the ADGRL1 fragments on the cell surface behave as independent proteins. The fragments are independently distributed at rest [16,25], and their distribution can be independently modified by antibodies that patch the NTF together while leaving the CTF untouched [16,25]. A similar behavior is displayed by other AGPCRs like ADGRE2 (formerly EMR2), where, upon binding a ligand, the NTF translocates into lipid rafts where it reassociates with the CTF, generating an intracellular signal [32].

Our current study confirms and extends these observations. Figure 5 demonstrates that in untreated cells stably expressing ADGRL1, its fragments are poorly colocalized on the cell surface (Figure 5, Figure 6b and Figure S2a) and undergo separate recycling (Figure 5). The CTF is especially prone to endocytosis; to a large extent it is located in the endocytic compartments, presumably End and PNC. This makes the plasma membrane rich in the NTF, which remains on the cell surface without attaching to the CTF. A strong ligand of the NTF, LTX^N4C^, induces redistribution of both fragments on the cell surface and increases their colocalization (Figure 5, Figure 6 and Figure S2), which is known to trigger CTF-mediated signaling to Ca^2+^ stores [16]. It is also interesting that ligand binding to the cell-surface NTF induces the translocation of the CTF from endocytic compartments back to the cell surface (Figure 5, Figure 6 and Figure S2), suggesting that an activated NTF may produce some intracellular signals that indicate a physiological requirement for the CTF to reassociate with the ligand–NTF complex, form a complete receptor, and activate the ADGRL1 signaling cascade.

Additionally, our experiments show that PFOA (an amphiphilic perfluorinated short-chain carboxylic acid) can separate the NTF from CTF without solubilizing or damaging the membrane and without inhibiting toxin binding to the NTF (Figure 2, Figure 3 and Figure 4). Short treatment with a low concentration of PFOA substantially changes the independent distribution of the ADGRL1 fragments on the cell surface, significantly decreasing their colocalization, even if they are held together by the ligand, LTX^N4C^ (Figure 5, Figure 6 and Figure S2). The separation of the fragments facilitates their separate recycling (Figure 5 and Figure S2c). A large proportion of the CTF is then promptly endocytosed (Figure 5), possibly as a result of its activation upon the NTF removal, as has been demonstrated by ultrastructural studies for other AGPCRs [30,31]. Activation-induced (agonist-induced) modifications (e.g., phosphorylation and altered intracellular interactions), leading to ligand-induced endocytosis and subsequent recycling or degradation, are a known feature of many GPCRs [43,44,45,46,47].

It is important to note that PFOA not only triggers the dissociation of ligand-free receptors but also inhibits the reassociation of the LTX^N4C^-bound NTF with the CTF (Figure 5, Figure 6 and Figure S2). This is expected to disable LTX^N4C^-induced intracellular signaling and cause PFOA-dependent inhibition of the LTX^N4C^-induced neurotransmitter release. Indeed, PFOA inhibits the toxin action quickly (Figure S4) and with micromolar affinity (Figure 7 and Figure S3b), suggesting that it acts extracellularly and does not strictly require a redistribution of receptor fragments within the membrane or their internalization. Fragments’ internalization is likely to be a consequence of PFOA-induced inability of the fragments to reassociate and self-activation of the CTF (see below). Also, the preservation, in the presence of submaximal PFOA, of the principal mechanism of LTX^N4C^ action (burst-like exocytosis) (Figure 7 and Figure S3) indicates that the toxin is still able to trigger the same type of intracellular signaling, but requires more time to achieve the same level of signal. This implies that PFOA decreases the number of functional NTF-CTF complexes available for activation.

In conclusion, the results of our experiments with PFOA strongly support the hypothesis that LTX^N4C^ stimulates exocytosis of neurotransmitters through reassociated fragments, while their dissociation inhibits the toxin’s effect.

**Figure 8:**
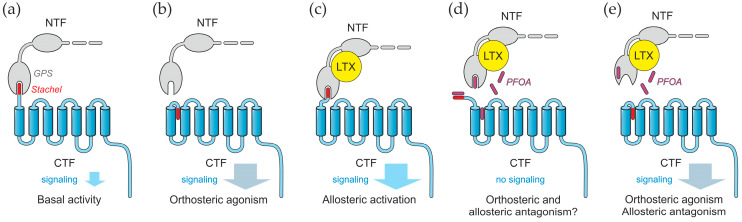
A generalized structure of AGPCRs and the proposed mechanisms of their modulation. (**a**) When the receptor is cleaved into fragments, which remain in complex, it demonstrates basal (low) signaling. (**b**) Upon the dissociation of the fragments, the hydrophobic *stachel* peptide interacts with the 7TMR cavity and, acting as a tethered ligand, strongly activates the receptor (orthosteric agonism). (**c**) We propose that strong ligands like αLTX or LTX^N4C^ bind to the NTF, promoting the reassociation and increased interaction between the NTF and CTF. This leads to a ligand-specific type of signaling through allosteric agonism. Note that this type of signaling is likely different from that in (**b**). (**d**,**e**) Our current findings suggest that PFOA directly interacts with one or both ADGRL1 fragments and thereby facilitates their dissociation, blocking the LTX^N4C^-induced signaling (i.e., acting as an allosteric antagonist). PFOA could act through several distinct mechanisms, which could be experimentally differentiated based on their unique impacts on receptor signaling. (**d**) As an amphiphilic molecule, PFOA could occupy all or some of the hydrophobic binding sites present on the receptor fragments: the hydrophobic pocket in the GPS domain, the 7TMR cavity, and/or the surface of *stachel* peptide itself. Due to its structural differences from *stachel*, PFOA is unlikely to activate the GPCR in the same manner and may function as an orthosteric antagonist. (**e**) Alternatively, PFOA could act allosterically on the GPS domain, causing it to release *stachel*, which could then occupy the orthosteric site (the 7TMR cavity) and trigger a strong intracellular signal.

Our results could also contribute to the understanding of AGPCR signaling mechanisms and the role of LTX^N4C^ in ADGRL1 activation. The unique structure of these receptors (their cleavage into the two fragments, the tight non-covalent bond between the NTF and CTF, and the ability of the two fragments to dissociate) has been actively studied in recent years [29,48,49,50]. Many aspects of the AGPCR signaling mechanism are still unclear, but it is thought that the *stachel* peptide may serve as a tethered ligand [48,51] of the CTF, which is itself a proper GPCR (Figure 8a,b).

When the fragments dissociate (for example, through mechanical force), the *stachel* peptide comes out of the pocket in the GPS domain. Its hydrophobic nature causes *stachel* to bury itself within the hydrophobic environment of the 7TMR cavity [31,50,52], and this hyperactivates the receptor (orthosteric agonism) [31,52,53] (Figure 8b). Experiments have also shown that, due to conserved structures of the GPS domain and *stachel*, peptides derived from the *stachel* sequence of one AGPCR group can promiscuously activate AGPCRs of different families [48]. Moreover, distinct AGPCRs are able to exchange their fragments on the cell surface, creating chimeric receptors with a mosaic of ligand binding and signaling features [25].

The binding of different (soluble or extracellular matrix- or membrane-bound) ligands induces conformational changes in the NTF (Figure 8c), and this triggers a certain response through the CTF linked to variable downstream pathways [54]. Which specific pathway is activated may depend on the nature of the ligand (allosteric agonism) [55]. LTX^N4C^ and αLTX (through one of its two actions) activate the receptor in this manner, stimulating the inositol trisphosphate (IP_3_) signaling cascade and an increase in intracellular Ca^2+^ levels, which triggers “spontaneous” (non-synchronized) vesicular exocytosis [9,16].

Our findings suggest that PFOA acts by binding quickly and specifically to one or both of the ADGRL1 fragments, prompting their dissociation. The exact binding mechanism is unclear, but two verifiable scenarios can be proposed (Figure 8d,e). According to the first scenario, amphiphilic PFOA may bind to hydrophobic sites on the receptor, such as the GPS domain pocket or the 7TMR cavity (Figure 8d). Based on the IC50 values (Figure 7c,e), this binding has a relatively high affinity (K_D_ ~ 1 μM) and likely involves both hydrophobic and polar interactions, much like PFOA’s main binding site on human serum albumin, the principal PFOA-binding protein in the blood [37]. These actions of PFOA are expected to inhibit both the NTF-CTF reassociation (allosteric antagonism) and *stachel*’s positioning in the orthosteric site (orthosteric antagonism). PFOA could also form a complex with *stachel*, protecting it from water (Figure 8d).

An alternative scenario can be modeled on the ability of PFOA to bind β-lactoglobulin and induce substantial changes in its secondary structure, which result in (i) the formation of a new, energetically favorable pocket for PFOA and (ii) the allosteric inhibition of the binding site for a hydrophobic dye [56]. This model proposes that PFOA could bind to a site on the GPS domain and induce a conformational change, which impairs the *stachel*-binding pocket and releases the CTF (Figure 8e). The CTF could then undergo orthosteric activation by *stachel*. These two models of PFOA action can be distinguished on the basis of downstream effects. In both cases, PFOA would block the effect of LTX^N4C^, but the intracellular signal triggered by the free CTF is expected to be different when the 7TMR cavity is occupied by PFOA or the tethered ligand (*stachel*). The two proposed models of PFOA action on AGPCR fragments, outlined above, are currently hypothetical and necessitate direct validation through ultrastructural techniques.

Together, our findings demonstrate that the biological functions of ADGRL1, a protein that is highly expressed in the brain and peripheral neurons [24], can be perturbed by even small concentrations of PFOA. Although its functions are only beginning to be revealed, it is clear that ADGRL1 plays an important role in the nervous system. For example, ADGRL1 knockout in mice, despite powerful compensatory mechanisms, results in hyperactivity, obligatory parental infanticide, inability to process sensory information, and impairments of synaptogenesis [57]. In humans, ADGRL1 haploinsufficiency is associated with cranial malformations, attention deficit/hyperactivity disorder, developmental delay, autism spectrum disorders, intellectual disability, and epilepsy [57]. If PFOA affects ADGRL1 in real-world conditions, this may be associated with an increased risk of neurological disorders.

PFOA is a commonly used industrial chemical [58,59] that has been found to accumulate in the environment and in humans [60,61] at concentrations similar to those in this study [62]. For instance, manufacturing workers in Decatur, AL, in 1998 had an average PFOA blood level of 899 μg/L [62], equivalent to 2.17 μM. At this concentration, PFOA is estimated to inhibit 60–70% of LTX^N4C^-induced exocytosis, which may affect the normal functions of ADGRL1, leading to serious neurological and mental issues, similar to those mentioned above. For comparison, research indicates that PFOA exposure in adult mice is linked to behavioral changes, such as reduced habituation and increased activity [63,64]. Neonatal PFOA exposure has also been associated with immune system changes which, under certain genetic predispositions, may contribute to symptoms related to autism and attention deficit/hyperactivity disorder [65]. It is possible that at least some of these adverse effects of PFOA were mediated by ADGRL1.

How serious is the risk? Due to concerted efforts of multiple US agencies, PFOA levels in blood have been steadily declining from 0.013 μM (1999–2000) to 0.004 μM (2017–2018) in the general population and from 0.55 μM (2005–2006) to 0.004 μM (2018) in exposed communities [62]. Although PFOA can cross the blood–brain barrier, its levels in the brains of Australian dementia patients (0.002 μM) were 8–10-fold lower than in the serum (0.016 μM) [66], indicating a potentially low risk of neurological disorders and ADGRL1 dysfunction. However, a recent Italian study of an exposed group reported much higher PFOA concentrations both in the serum (0.524 μM) and in the brain (0.033–0.38 μM; average 0.138 μM) [67], which falls within the effective range of PFOA for ADGRL1 dissociation.

This demonstrates that PFOA still poses a substantial risk for the functions of ADGRL1 and other neuronal proteins, especially in a developing nervous system. To mitigate this risk, it may be possible to use the ADGRL1-LTX^N4C^ system, described here, as a sensitive reporter assay for detecting PFOA and other neurologically active contaminants to prevent exposure. The system can also be used for in-depth studies of the signaling mechanisms downstream of the αLTX receptor. Reciprocally, the system can be employed for testing various low-molecular-mass substances for their ability to inhibit or activate ADGRL1 with the aim of designing ADGRL1-specific medicines with potential neurological applications.

## 4. Materials and Methods

### 4.1. Materials

All reagents and chemicals were from Merck (Merck Life Science UK Limited, Gillingham, Dorset, UK), unless otherwise stated. Antibodies used were as follows: rabbit affinity-purified polyclonal anti-NTF IgG RL1 [12,22], rabbit polyclonal anti-CTF anti-serum R4 [16], rabbit anti-NRX anti-serum [12], mouse anti-V5 monoclonal antibody V5-10, rabbit anti-αLTX serum [68], IRDye^®^ 800CW goat anti-rabbit IgG secondary antibody and IRDye^®^ 680RD goat anti-mouse IgG secondary antibody (LI-COR Ltd. United Kingdom, Cambridge, UK), rabbit anti-V5 polyclonal antibody (to stain the NTF of the ADGRL1 construct used), mouse monoclonal ant-*myc* antibody 9E10 (to stain the CTF), goat anti-rabbit IgG secondary antibody Alexa Fluor™ 488, and goat anti-mouse IgG secondary antibody Alexa Fluor™ 546 (Thermo-Fisher Scientific—UK, Life Technologies Limited, Paisley, UK). Dulbecco’s modified Eagle medium (DMEM), GlutaMAX^TM^, and Trypsin-EDTA were from Gibco (Life Technologies). Sterile water was from PAA (PAA Cell Culture Company, Cambridge, UK). The anti-Kv1.2 antibody used was kindly provided by J.O. Dolly (Imperial College London, London, UK) [69].

### 4.2. Solubilization and Affinity Chromatography of ADGRL1 Fragments

To prepare synaptosomes, rat cerebral cortices were homogenized in 0.32 M sucrose, 10 mM HEPES pH 7.4 at 4 °C, using a Potter S-type homogenizer (Potter-Elvehjem Homogeniser, Sartorius, Epsom, UK) at 900 rpm. The homogenate was centrifuged for 10 min at 4500 rpm. For further purification, the S2 supernatant was layered onto a step gradient consisting of 0.8 and 1.2 M sucrose in a centrifuge tube and centrifuged for 30 min at 45,000 rpm. The material concentrated at the 0.8–1.2 M sucrose interface was collected and centrifuged for 20 min at 20,000× *g*, yielding synaptosomes. To prepare the synaptic plasma membranes (SPM), the synaptosomes were quickly resuspended in a large volume of ice-cold 10 mM HEPES, pH 7.4, incubated for 1 h and centrifuged for 20 min at 20,000× *g*.

To compare ADGRL1 solubilization by different detergents, SPM pellets were resuspended in TBSE buffer (50 mM Tris-HCl, pH 7.6; 0.2 M NaCl; 2 mM EDTA;) containing protease inhibitors (104 mM AEBSF, 80 μM aprotinin, 4 mM bestatin, 1.4 mM E-64, 2 mM leupeptin, and 1.5 mM pepstatin A), phosphatase inhibitors cocktail 1 (Merck Life Science UK Limited), and increasing concentrations of Thesit or PFOA (as indicated in Results) and incubated for 2 h at 4 or 20 °C, with rotation. The solubilized membranes were centrifuged for 20 min at 20,000× *g*, yielding the supernatants and pellets used for Western blot analysis or WGA-affinity chromatography.

For Western blot analysis, proportional aliquots of the fractions were separated by SDS–polyacrylamide gel electrophoresis (SDS-PAGE) in reducing 8% gels as previously described [70]. Proteins were then electro-transferred onto a polyvinylidene difluoride (PVDF) membrane and immunostained with primary and secondary antibodies, using standard procedures.

For analytical affinity chromatography, the supernatants of SPM, solubilized in 2% Thesit or PFOA at room temperature (Load), were diluted with TBSE to 0.04% detergent and incubated with 50 μL of WGA–Sepharose 4B for 16 h at 4 °C. The liquid above the beads (Flow Through, FT) was collected, and the beads were washed with TBSE, containing a respective detergent, and eluted with hot SDS-PAGE sample buffer to ensure the complete removal of ADGRL1 from the gel matrix. The eluates were analyzed by Western blotting in parallel with proportional aliquots of the Load and Flow Through fractions.

### 4.3. Cell Culture

Mouse neuroblastoma NB2a clonal cells stably transfected with an ADGRL1 construct and multiply sorted by flow cytometry were kindly provided by Prof. K. Volynski (UCL Institute of Neurology, London, UK). The ADGRL1 construct (vL-Lm) contained a V5 epitope on the N-terminus and two *myc* epitopes on the C-terminus [25].

The NB2a cells were cultured in DMEM containing GlutaMAX^TM^ and 10% fetal bovine serum (FBS), at 5% CO_2_. To maintain ADGRL1 expression, the medium also contained 300 μg/mL G418 (Gibco). Cells were allowed to grow to 80% confluency and passaged every 2–3 days. To increase ADGRL1 surface expression, the cells were differentiated 24 h after plating by replacing the medium with a serum-free (SF) Neurobasal-A medium, containing 2% B-27 supplement (Thermo-Fisher Scientific) and 0.5 mM GlutaMAX^TM^, and incubated for 24–48 h, when they typically reached 70–80% confluence.

### 4.4. PFOA Treatment, LTX Binding, and Immunostaining of Cultured Cells

To analyze the effect of PFOA on toxin binding, the differentiated cells (expressing ADGRL1 or untransfected) were removed from the plates using 0.2 mM EDTA in PBS, washed by mild centrifugation, treated with 0–100 μM PFOA in SF medium for 20 min, and then incubated with/without 5 nM αLTX for 20 min. The cells were then centrifuged; the resulting supernatants and cells were analyzed by immunoblotting with antibodies against the NTF (anti-V5) and αLTX.

To assess the effect of PFOA on cell viability, the ADGRL1-expressing or untransfected cells were seeded into 96-well plates with clear bottom (Corning^®^, Thermo-Fisher Scientific—UK, Life Technologies Limited) at a density of 10 × 10^3^ cells per well, in DMEM containing GlutaMAX^TM^ and 10% FBS. After 24 h, DMEM was changed to SF medium, and the cells were differentiated for 24–48 h, reaching ~20 × 10^3^ cells per well. The cells were then treated with 0–100 μM PFOA for 4 and 24 h and assayed for viability using an MTT Cell Growth Assay Kit (Merck Life Science UK Limited) and a VersaMax microplate reader (Molecular Devices, LLC, San Jose, CA, USA).

PFOA toxicity was also tested in a PI-based cell death assay adapted from a published method [40,41]. Cells were allowed to grow and differentiate in clear-bottomed 96-well plates as described above and then incubated with different concentrations of PFOA in SF medium for 24 h. The cells were washed with PBS and incubated for 10 min with 200 μL PBS containing 60 μM PI (a cell-impermeant DNA-binding fluorescent dye, for staining dead cells) (Thermo-Fisher Scientific) and 15 μM Vybrant^TM^ DiO (a lipophilic fluorescent dye, for staining all cells in a well) (Thermo-Fisher Scientific). Dye stocks were prepared according to the manufacturer’s protocols. The cells were then washed and their fluorescence measured in a Fluoroskan Ascent FL microplate fluorometer (Labsystems, Thermo-Fisher Scientific) using the following excitation/emission filter pairs: 485/538 nm (DiO) and 530/635 nm (PI). The PI fluorescence signal was then normalized to the DiO fluorescence for each well. As a negative control, the cells were incubated in SF medium not containing PFOA, while the maximum response was elicited by incubating cells with 0.1% SDS.

To assess the impact of 100 μM PFOA on cells’ ability to proliferate, differentiated ADGRL1-expressing cells (~2 × 10^6^ per 75 mL culture flask) were incubated overnight in sterile SF medium containing 0 or 100 uM PFOA and then washed and allowed to recover for 8 h in the complete medium (to remove traces of PFOA due to serum albumin binding). The cells were then harvested using Versene (Thermo-Fisher Scientific) without trypsin, replated into 175 mL culture flasks in the complete medium, which was replaced 4 h later with SF medium, and allowed to grow. After 24 and 48 h, the cells (3–4 × 10^6^) were harvested, centrifuged, resuspended in 30 mL PBS, and then counted using a hemocytometer.

For fluorescent immunostaining, the ADGRL1-expressing or untransfected NB2a cells were grown on poly-D-lysine-coated glass coverslips, differentiated overnight in SF medium, treated for 20 min with/without 100 μM PFOA in SF medium, incubated with/without 5 nM LTX^N4C^ for 20 min, and then washed and fixed with 2% formaldehyde. The cells were then permeabilized, blocked in 10% goat serum, and incubated overnight at 4 °C with anti-V5 and anti-*myc* primary antibodies (1:2000 and 1:1000 dilution, respectively). After an extensive wash, the primary antibodies were detected with secondary antibodies conjugated with Alexa Fluor^TM^ 488 and 546, mounted on glass slides, and imaged under a confocal microscope.

### 4.5. Confocal Microscopy

The mounted cell preparations were imaged using a DMI8-CS laser scanning confocal microscope (Leica Mikrosysteme Vertrieb GmbH, Wetzlar, Germany) equipped with a 63x/1.4 oil immersion objective and controlled by the Leica Application Suite software LAS X 2.0.0.14332 (Leica Mikrosysteme Vertrieb GmbH). The following configurations were used in double-staining experiments: excitation, 488 and 561 nm; emission, 505–530 and >580 nm. Images were acquired by scanning 1 μm thick confocal sections near the cell’s equator, using the same microscope settings for all the samples. Multicolor images (1024 × 1024 pixels) were saved as TIFF files. The fluorescence of individual fluorophores was measured in separate channels for each cell, and both signals were adjusted to their dynamic ranges.

The images were preprocessed (background removal and deconvolution) and analyzed using the Fiji software suite release 2.16.0 (Laboratory for Optical and Computational Instrumentation, Madison, WI, USA) [71]. For measuring the NTF-CTF colocalization in the plasma membrane, 1 μm thick optical sections were acquired near the cell’s equator. Linear regions of interest (ROIs) were then created along the plasma membrane using the Weka Segmentation 3.8.6 plugin [72]. The distribution profiles of the two fluorescent signals along these ROIs were plotted as a function of distance along the membrane circumference for illustration (Figure 6a) and compared by pixel intensity correlation analysis (Figure 6b).

To quantify the fragments’ distribution between the membrane and cytosol, the preprocessed images were segmented and masked using the Weka Segmentation software; fluorescent signals were integrated for both segments and normalized to the total fluorescent signal in each channel (Figure 6c,d).

Fragments’ colocalization in whole-cell images was quantified by pixel intensity correlation analysis using Colocalization Finder and Coloc 2 plugins (Figure S2). Different correlation parameters were used to assess the degree of colocalization, Pearson’s correlation coefficient r, Manders’ split colocalization coefficients (M_1_ and M_2_) [73], and Li’s Intensity Correlation Quotient (ICQ) [74], whereas the statistical significance of the colocalization was evaluated by calculating the Costes’ probability [42].

### 4.6. Neurotransmitter Release

Spontaneous synaptic activity at mouse NMJs was recorded using flexor digitorum brevis neuromuscular preparations from the hind paws of 3–6-week-old male mice (Charles River Laboratories, Margate, UK). The experiments were planned and conducted in accordance with the ARRIVE guidelines for reporting animal experiments [75,76], which are also applicable to ex vivo tissues used here. To ensure sample randomization, the two muscles obtained from each animal were used under different experimental conditions, with each PFOA concentration applied to tissues collected from three different animals. Given the clear differences observed in control and treated animals, the sample size was minimized, while ensuring robust statistics, by using 3–6 muscles chosen randomly for each data point (*n* = 3–6) and recording from 5 to 10 myofibers for each condition (*N* = 15–30). In addition to dedicated control preparations, control recordings were carried out on each muscle prior to PFOA addition. The muscles were pinned to the bottom of Petri dishes coated with Sylgard (Dow Silicones UK Ltd., Barry, Wales, UK). The preparation was observed under a binocular microscope and perfused with oxygenated physiological buffer (137 mM NaCl; 5 mM KCl; 1 mM MgCl_2_; 0.2 mM EGTA; 5.6 mM glucose; 10 mM HEPES; pH 7.5). The preparations were treated for 20 min with 0–100 μM PFOA and then stimulated with 0.25 nM LTX^N4C^. The postsynaptic membrane potential (Vm) was measured using sharp glass microelectrodes filled with 5 M ammonium acetate. The signals were pre-amplified using an Axoclamp 2B amplifier (Molecular Devices, LLC), and then further amplified and filtered by a differential amplifier (LPF202A, Warner Instruments, Harvard Apparatus, Holliston, MA, USA) and a harmonic frequency quencher (HumBug, Digitimer Ltd., Welwyn Garden City, Hertfordshire, UK). The analog signals were then digitized with a Digidata 1322A digitizer controlled by AxoScope 10.7 software (Molecular Devices, LLC). MEPPs were automatically detected in the recorded traces using Mini Analysis software version 6.0.7 (Synaptosoft Inc., Decatur, GA, USA); the data were blindly analyzed by two researchers, and no data points were excluded.

### 4.7. Statistical Analysis

Statistical analysis was performed in Prism 6 software (GraphPad Software, Boston, MA, USA). For comparisons between two groups of data, the two-tailed Student’s *t*-test or the Mann–Whitney U Test was performed. For three or more group comparisons, one-way analysis of variance (ANOVA) with Bonferroni correction was applied. Statistical significance was accepted at *p* < 0.05. The level of significance is indicated on the graphs (*, *p* < 0.05; **, *p* < 0.01; ***, *p* < 0.001: #, *p* < 0.0001).

## Data Availability

The original contributions presented in this study are included in the article/Supplementary Material. Further inquiries can be directed to the corresponding author(s).

## Supplementary Materials

The following supporting information can be downloaded at https://www.mdpi.com/article/10.3390/toxins17070359/s1, Figure S1: Low concentrations of PFOA are not toxic to cells.; Figure S2: Changes in the NTF and CTF colocalization induced by PFOA; Figure S3: PFOA does not modify the intrinsic mechanisms of LTXN4C-induced acetylcholine exocytosis; Figure S4: PFOA inhibits LTXN4C-induced acetylcholine exocytosis when added after toxin.
