# Supplementary material for: The Dissociation of Latrophilin Fragments by Perfluorooctanoic Acid (PFOA) Inhibits LTXN4C-Induced Neurotransmitter Release
Source: Toxins (Basel). 2025 Jul 20;17(7):359. doi: 10.3390/toxins17070359 (PMC12300799; doi:10.3390/toxins17070359)
Supplement: Supplementary file 1 [file toxins-17-00359-s001.zip › toxins-3751210-supplementary.pdf]

toxins

MDPI

Supplementary Material

The Dissociation Of Latrophilin Fragments by Perfluorooctanoic Acid Inhibits LTX^{N4C}-Induced Neurotransmitter Release

Evelina Petitto, Jennifer K. Blackburn, M. Atiqur Rahman and Yuri A. Ushkaryov

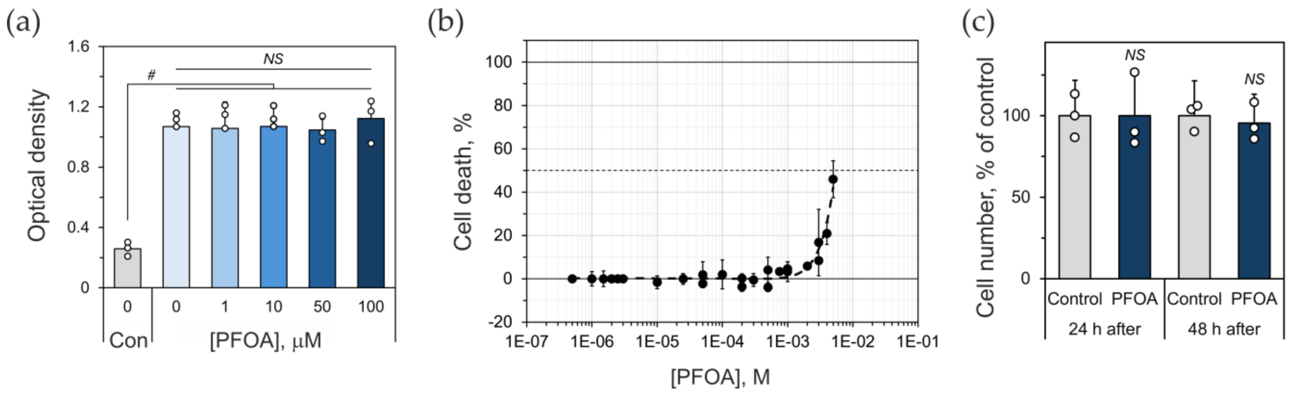

Figure S1. Low concentrations of PFOA are not toxic to cells. The cells were incubated with the specified concentrations of PFOA for 24 h and assayed for viability using an MTT test (**a**) or a PI-based cell death assay (**b**). At the time of assay, the number of cells per well reached ~30,000, except in control, where no cells were added. In (**a**), the graph bars are the means ± SE; open circles, the means from individual experiments (n = 3), with 4 replicates in each (N = 12). In (**b**), the symbols represent the means ± SD; the number of independent experiments n = 3, with 3 replicates in each (N = 9). (**c**) The ability of cells to proliferate after 16 h of treatment with 100 µM PFOA. PFOA was removed, and cells were allowed to grow for 24 h or 48 h (as described in Section 4.4). Cell numbers are expressed as per cent of control (untreated) cells; the data are the means ± SD from n = 3 experiments, with 3 replicates (N = 9). The symbols denote the statistical significance: #, $p < 0.001$; *NS*, non-significant. Note that the incubation with up to 100 µM PFOA does not affect the viability of cells or their ability to proliferate, and does not cause cell death.

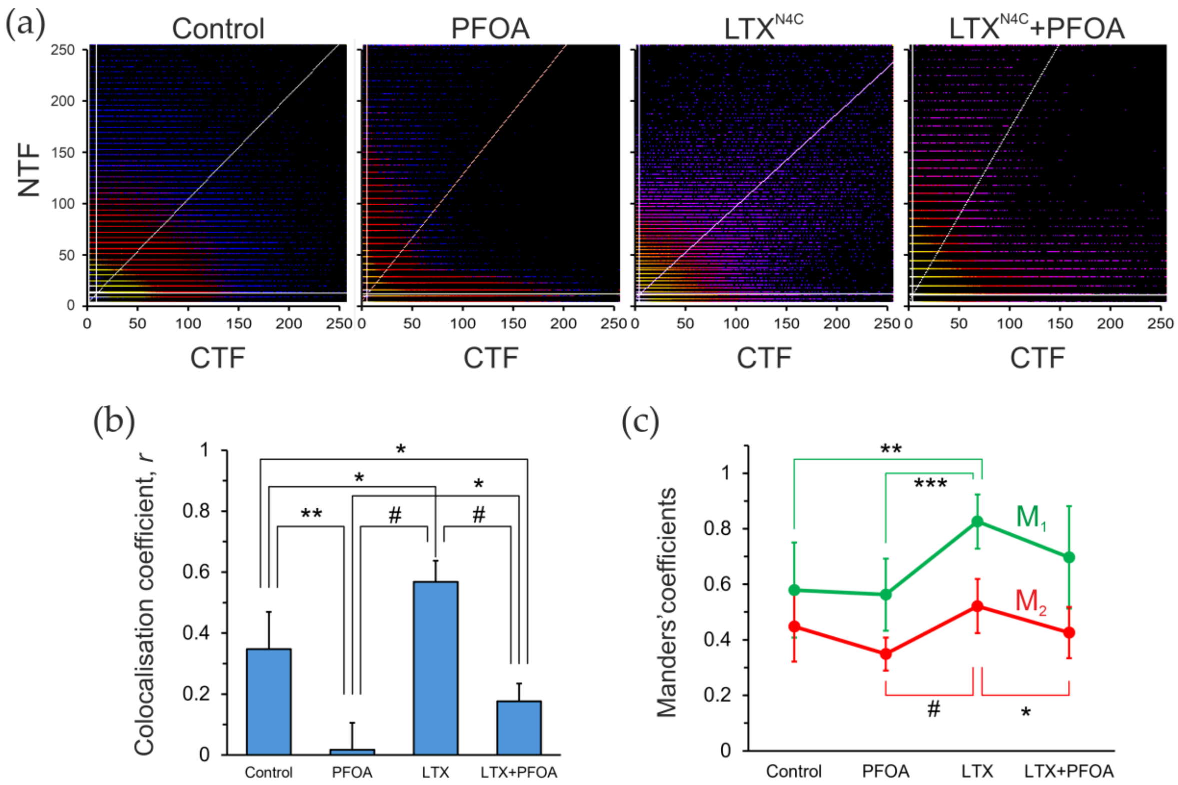

Figure S2. Changes in the NTF and CTF colocalization induced by PFOA. (**a**) Representative colocalization scatter plots of whole-cell confocal images as in Figure 5a. The lines show two background thresholds and a linear regression solution. (**b**) Corresponding changes in the Pearson's correlation coefficient, *r*. (**c**) Changes in Manders' split coefficients M_1 and M_2. M_1 indicates the fraction of NTF fluorescence that is located in pixels where some intensity of CTF fluorescence is also present; reciprocally, M_2 shows the fraction of CTF fluorescence in those pixels that also contain some NTF fluorescence. Note that M_1 is always bigger than M_2, consistent with a large proportion of the CTF located inside the cell and not colocalized with the NTF. The statistical significance of differences: *, $p < 0.05$; **, $p < 0.01$; ***, 0.001; #, $p < 0.0001$; the differences between other values are nonsignificant; the number of experiments was $n = 3$, the numbers of replicates were: N =30 in control; $N = 15$ in PFOA experiments; $N = 23$ in LTX^{N4C} experiments; $N = 30$ in LTX^{N4C}+PFOA experiments.

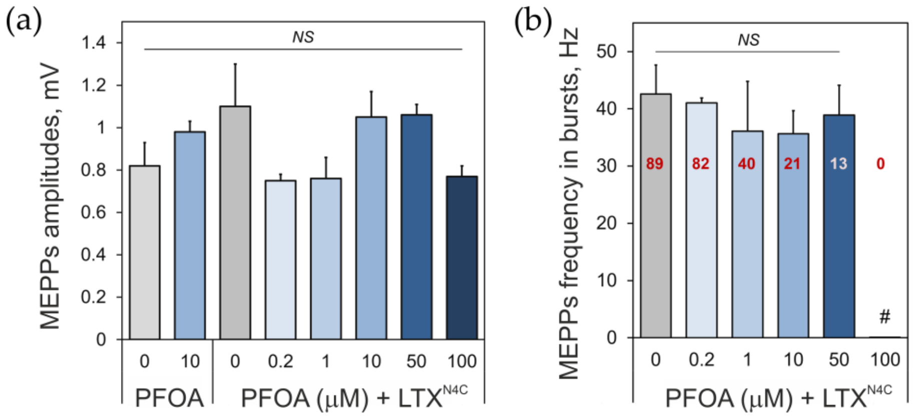

Figure S3. PFOA does not modify the intrinsic mechanisms of LTX^{N4C}-induced acetylcholine exocytosis. (**a**) The average amplitude of individual MEPPs is not affected by 0.2–100 μM PFOA or LTX^{N4C}. The number of experiments n = 3–6, with N = 16–32 individual synapses recorded. (**b**) The average frequency of MEPPs in LTX^{N4C}-induced bursts does not change in the presence of 0.2–100 μM PFOA, which only decreases the number of bursts and then fully blocks their occurrence. The numbers indicate the per cent of NMJs tested that showed any LTX^{N4C}-induced bursts of MEPPs.

The number of experiments was $n = 3$, the number of individual synapses recorded $N = 25$–44. All bars are the means ± SE; the statistical significance compares all values to control in the absence of both PFOA and LTX^{N4C} in (**a**), and the effects of LTX^{N4C} in the presence of PFOA to that in its absence in (**b**); #, $p < 0.0001$; *NS*, non-significant.

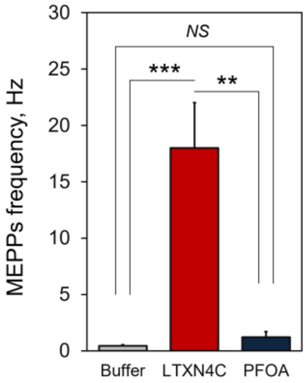

Figure S4. PFOA inhibits LTX^{N4C}-induced acetylcholine exocytosis when added after toxin. The average frequency of MEPPs under control conditions, after the onset of the effect of 0.25 nM LTX^{N4C}, and after the addition of 100 μM PFOA. The bars are the means ± SE; the statistical significance compares the values as indicated: **, $p < 0.01$; ***, 0.001; *NS*, nonsignificant; $n = 3$ independent experiments, $N = 19$ cells recorded after LTX^{N4C} addition and 18 after subsequent PFOA addition. Note that PFOA significantly inhibits exocytosis previously triggered by the LTX^{N4C}-induced activation of ADGRL1.
